# Clinical utility of metagenomic next-generation sequencing in the diagnosis of invasive pulmonary aspergillosis in acute exacerbation of chronic obstructive pulmonary disease patients in the intensive care unit

**DOI:** 10.3389/fcimb.2024.1397733

**Published:** 2024-07-12

**Authors:** Siqiang Niu, Dezhi Liu, Yan Yang, Limin Zhao

**Affiliations:** ^1^ Department of Respiratory and Critical Care Medicine, Zhengzhou University People's Hospital, Zhengzhou, China; ^2^ Henan Provincial People’s Hospital, Zhengzhou, China; ^3^ Xinxiang City Central Hospital, Xinxiang, Henan, China; ^4^ Department of Respiratory and Critical Care Medicine, Henan Provincial People’s Hospital, Zhengzhou, China; ^5^ Zhengzhou University People’s Hospital, Zhengzhou, Henan, China; ^6^ Henan University People’s Hospital, Zhengzhou, Henan, China

**Keywords:** invasive pulmonary aspergillosis, diagnosis, acute exacerbation of chronic obstructive pulmonary disease, metagenomic next-generation sequencing, traditional tests, GM test

## Abstract

**Objective:**

To explore the clinical utility of metagenomic next-generation sequencing (mNGS) in diagnosing invasive pulmonary aspergillosis (IPA) among patients with acute exacerbation of chronic obstructive pulmonary disease (AECOPD) in the intensive care unit (ICU).

**Methods:**

A retrospective analysis was conducted on patients with AECOPD admitted to the ICU of Xinxiang Central Hospital in Henan Province, China, between March 2020 and September 2023, suspected of having IPA. Bronchoalveolar lavage fluid (BALF) samples were collected for fungal culture, the galactomannan (GM) test, and mNGS. Based on host factors, clinical features, and microbiological test results, patients were categorized into 62 cases of IPA and 64 cases of non-IPA. Statistical analysis was performed to compare the diagnostic efficacy of fungal culture, the serum and BALF GM test, and mNGS detection for IPA in patients with AECOPD.

**Results:**

1. The sensitivity and specificity of mNGS in diagnosing IPA were 70.9% and 71.8% respectively, with the sensitivity of mNGS surpassing that of fungal culture (29.0%, *P*<0.01), serum GM test (35.4%, *P*<0.01), and BALF GM test (41.9%, *P*<0.05), albeit with slightly lower specificity compared to fungal culture (90.6%, *P >*0.05), serum GM test (87.5%, *P >*0.05), and BALF GM test (85.9%, *P >*0.05).Combining fungal culture with the GM test and mNGS resulted in a sensitivity of 80.6% and a specificity of 92.2%, underscoring a superior diagnostic rate compared to any single detection method. 2.mNGS accurately distinguished strains of the *Aspergillus* genus. 3.The area under the ROC curves of mNGS was 0.73, indicating good diagnostic performance. 4.The detection duration for mNGS is shorter than that of traditional fungal culture and GM testing.

**Conclusion:**

mNGS presents a pragmatic and highly sensitive approach, serving as a valuable complementary tool to conventional microbiological tests (CMT). Our research demonstrated that, compared to fungal culture and GM testing, mNGS exhibits superior diagnostic capability for IPA among patients with AECOPD. Integration of mNGS with established conventional methods holds promise for improving the diagnosis rate of IPA.

## Introduction

Chronic obstructive pulmonary disease (COPD) is a chronic, non-specific inflammatory airway disease characterized by persistent and progressively worsening airflow obstruction and alveolar destruction. The 2023 Global Initiative for Chronic Obstructive Lung Disease (GOLD) version defines acute exacerbation of COPD(AECOPD) as an acute event characterized by deterioration in dyspnea and/or cough with sputum production among patients with COPD, occurring within 14 days, possibly accompanied by tachypnea and/or tachycardia (Zantah et al., 2020). In the ICU, certain critically ill patients are often subjected to prolonged courses of broad-spectrum antibiotics, glucocorticoids, invasive mechanical ventilation, and central venous catheterization (Huang et al., 2017). These factors collectively contribute to a mounting incidence of IPA among patients experiencing AECOPD, significantly compromising their prognosis and survival duration. Literature reports indicate that over 10% of COPD patients develop IPA, with mortality rates ranging from 50% to 100% when COPD is compounded by IPA. Therefore, there is an urgent need for early diagnosis and treatment of IPA.

Currently, common diagnostic methods for aspergillosis include fungal culture, the (1,3)-β-D glucan (G test), and the galactomannan (GM) test. However, these methods exhibit certain limitations, such as time-consuming procedures and low diagnostic rates. Additionally, the clinical manifestations of IPA lack specificity, leading to a decreased rate of diagnosis and delayed antifungal treatment, ultimately resulting in elevated mortality rates.Therefore, early diagnosis of IPA is essential.

The rapid advancements in genome sequencing technologies and bioinformatics approaches offer robust solutions to tackle these clinical diagnostic challenges. mNGS emerges as a promising microbial identification technique. It operates independently of culture and hypotheses, serving as a broad-spectrum sequencing method capable of circumventing the limitations of existing diagnostic tests. It facilitates direct pathogen detection without reliance on primers or probes. While mNGS has found widespread application in diagnosing microbial pathogens, its clinical utility in pulmonary aspergillosis remains relatively unexplored (Denning, 2015; Bao et al., 2022). Previous research findings predominantly stem from case reports and small case series, providing constrained clinical insights and evidence.

In this study, we investigated patients with AECOPD complicated by IPA. We compared the diagnostic efficacy of mNGS with that of fungal culture, as well as that of serum and BALF GM tests.

## Materials and methods

### Study design and participants

**Figure 1:**
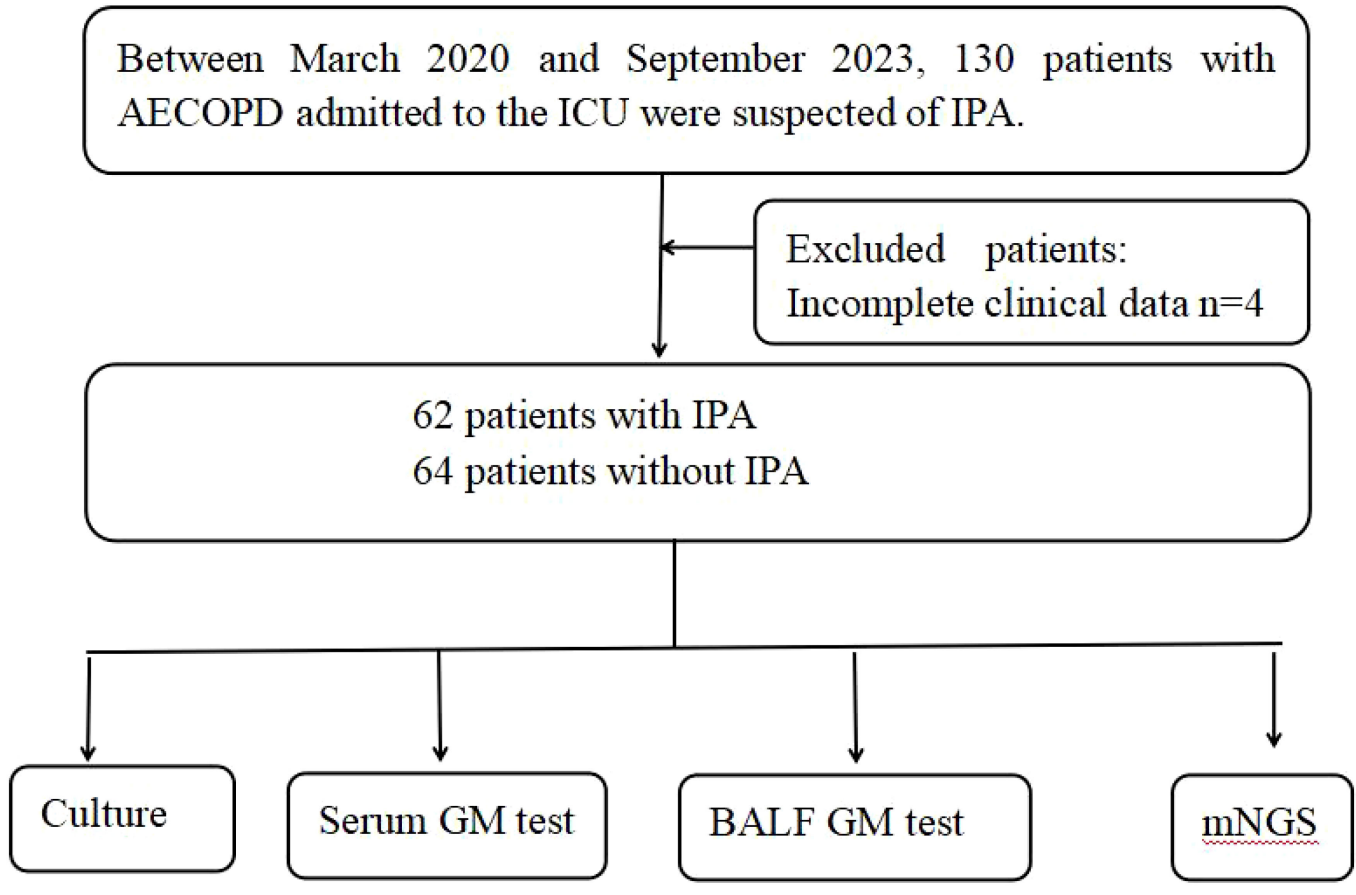
Flowchart of case selection.

This is a retrospective study involved patients with AECOPD admitted to the ICU of the Xinxiang Central Hospital between March 2020 and September 2023. Eligible patients were those suspected of having IPA, meeting specific criteria: (1) presence of host factors: such as prolonged corticosteroid use exceeding 3 weeks, AECOPD, extended ICU stay, prolonged mechanical ventilation, indwelling catheters, total parenteral nutrition, and prolonged broad-spectrum antibiotic usage; (2)Primary clinical characteristics, including: a. Fever persisting for >96 hours with no improvement despite aggressive antibiotic therapy. b. Symptoms and signs of pulmonary infection. c. Chest CT showing lesions such as “halo sign” and “crescent sign”. (3) completion of bronchoalveolar lavage; (4) availability of culture, GM, and mNGS results (Russo et al., 2011; Otu et al., 2023). The final diagnosis was established based on the patients’ host factors, clinical characteristics, and microbiological test results, the final diagnosis was established. These patients were suspected of IPA, with a total of 126 patients included (62 with IPA and 64 without IPA) (**Figure 1**).

This study was approved by the Ethics Committee of Xinxiang city Central Hospital, Henan Province, China. Individual consent for this retrospective analysis was waived.

### Data collection

Demographic and clinical data were extracted from electronic medical records (EMRs), including age, gender, diagnosis of underlying diseases, smoking history, dosage of steroids used, duration of antibiotic therapy, laboratory test results, characteristics during ICU treatment (such as length of ICU stay, duration of central venous catheterization, SOFA score, duration of mechanical ventilation, rapid shallow breathing index (RSBI), etc.).

### Criteria of diagnosis of IPA and identification of pathogens

The diagnostic criteria for IPA were as follows: (1) Confirmed diagnosis: histopathology confirmed as fungi (independent of host factors and clinical features); or the presence of at least one host factor coupled with one major clinical feature or two minor clinical features, along with fungal identification in blood and secretions cultures. (2) Clinical diagnosis: simultaneous manifestation of at least one host factor, one major clinical feature, or two minor clinical features indicative of invasive pulmonary fungal disease, and microbiological evidence. (3) Possible diagnosis: simultaneous occurrence of at least one host risk factor, one major clinical feature, or two minor clinical features indicative of invasive pulmonary fungal disease (Ader et al., 2005; Tiew et al., 2022).

mNGS pathogen detection: BALF samples were obtained from all ICU-admitted patients within 48 hours of admission.While patients were under sedation, BALF samples were collected from the bronchial segment displaying severe lesions based on imaging findings. Each BALF sample had a minimum volume of 10 mL.Samples from the experimental group were dispatched to the Guangzhou Weiyuan Genomics Company for mNGS analysis.The specific steps were as follows: The BALF was processed using the Bioprep-24 biological sample homogenizer (/Tiangen Biotech Co., Ltd.China). Subsequently, 300μL of processed BALF was subjected to total DNA extraction using a DNA extraction kit. The extracted RNA was reverse transcribed into cDNA using reverse transcriptase (Thermo Fisher Scientific) and dNTPs, and DNA libraries were constructed. The prepared libraries underwent purification, amplification, and further purification. The concentration of the libraries was quantified using the Qubit 4.0 Fluorometer (Thermo Fisher Scientific).The libraries were then loaded onto the Illumina Nextseq CN500 sequencer for 75 single-end sequencing cycles. After sequencing, low-quality sequences were removed using Trimmomatic software to obtain high-quality data. The remaining microbial sequences were aligned to the microbial database constructed by Weiyuan Gene Company (Guangzhou, China). Subsequently, the aligned data were categorized and sorted according to viruses, bacteria, fungi, and parasites. Species-specific sequence reads (SSRN), relative abundances, identification confidences, and other information were obtained.

Traditional fungal culture: BALF specimens were inoculated onto Sabouraud’s dextrose agar plates and incubated in a fungal culture incubator at 28°C for 4–7 days. A fungal culture count of ≥10^5^ CFU/mL was deemed positive.

GM Test: The GM test employs a chemiluminescence method, wherein a GM concentration of ≤0.25 ug/L is deemed negative, while a concentration of ≥0.45 ug/L is considered positive. For concentrations ranging from 0.25 to 0.45 ug/L, results are considered indeterminate and should be evaluated comprehensively based on clinical findings.

### Evaluation criteria and observational indices

(1)Analyze the distribution of pathogens detected by mNGS and traditional *Aspergillus* culture<n.o></no> (2) The diagnostic efficacy of mNGS, traditional fungal detection, and combination testing was compared. The sensitivity, specificity, negative predictive value (NPV), positive predictive value (PPV) and J-index of mNGS, traditional fungal detection, the serum GM test, the BALF GM test,and combination testing were calculated and compared. (3) Finally, the time required for *Aspergillus* detection using mNGS, traditional fungal culture, and the GM test was compared.

### Statistical analyses

SPSS version 23.0 software was utilized for analysis. Normally distributed continuous data were expressed as mean ± standard deviation (x̅ ± s), and compared between groups employing independent sample t-tests.

## Results

### Clinical characteristics

During the study period, a total of 130 patients with AECOPD suspected of harboring IPA infection were enrolled. Four patients were excluded owing to incomplete clinical data, resulting in 126 patients being included in the final analysis, with 62 classified as patients with IPA and 64 as non-IPA patients. A comparison and summary of demographic and clinical characteristics between the two groups were conducted, encompassing gender, age, Body Mass Index(BMI), hypoalbuminemia, neutrophil (NEUT#)/lymphocyte (LYMPH#) ratio, revealing no significant differences between the IPA and non-IPA groups. The levels of the inflammatory factor IL-6 were significantly higher in the IPA group compared to the non-IPA group(*P* < 0.05). However, patients with AECOPD with a history of smoking, corticosteroid use, and antibiotic use exhibited a higher propensity for IPA development compared to non-IPA patients (*P* < 0.01). Throughout their ICU hospitalization, clinical indicators of both patient groups, such as length of ICU stay, duration of indwelling central venous catheter, duration of mechanical ventilation, rapid shallow breathing index, and incidence of septic shock, exhibited statistically significant differences (*P* < 0.05).The primary characteristics of all eligible patients are detailed in **Table 1**.

**Table 1:** Demographic and clinical profiles of the study cohort.

Characteristics	N	IPA	Non-IPA	*P*-value
Age(years)		54.45	53.39	0.39
Sex, n (%)				0.31
Male	60	27	33	
Female	66	35	31	
BMI		22.42 ± 3.50	23.02 ± 3.78	0.71
Smoking history(≥7 years) n %		26 (14.9%)	16 (25%)	0.029
Glucocorticoid usage (≥3 weeks)		34(54.8%)	14(21.9)	0.007
Duration of antibiotic use				0.01
≥14d	46	32	14	
<14d	80	30	50	
Diabetes mellitus, n (%)		28(45.2%)	(21.1%)	0.02
Laboratory findings at ICU admission, n (%)				
NEUT#/LYMPH#(≥0.9, <3.1 )		30 (46.9%)	26(40.6%)	0.464
Hypoalbuminemia<35g/L		28 (45.1%)	22(34.3%)	0.23
Interleukin(IL)-6>0.5ng/L		30(48.3%)	14(21.8%)	0.03
Disease severity at ICU admission, n (%)				
ICU stay>7d		32 (51.6%)	16(25%)	0.02
Central venous catheter placement >5d		29(47.9%)	15(23.1%)	0.02
SOFA score, median IQR		11(9,12)	9(8,11)	0.64
Invasive mechanical Ventilation>4d, (n)		31(50%)	15(43.8%)	0.04
Rapid shallow breathing index (RSBI)≤105, (n)		36 (58.1%)	22(33.3%)	0.001
Septic shock		34(54.8%)	17(26.5%)	0.001

### Classification of Aspergillus detected through mNGS and traditional fungal culture

**Figure 2:**
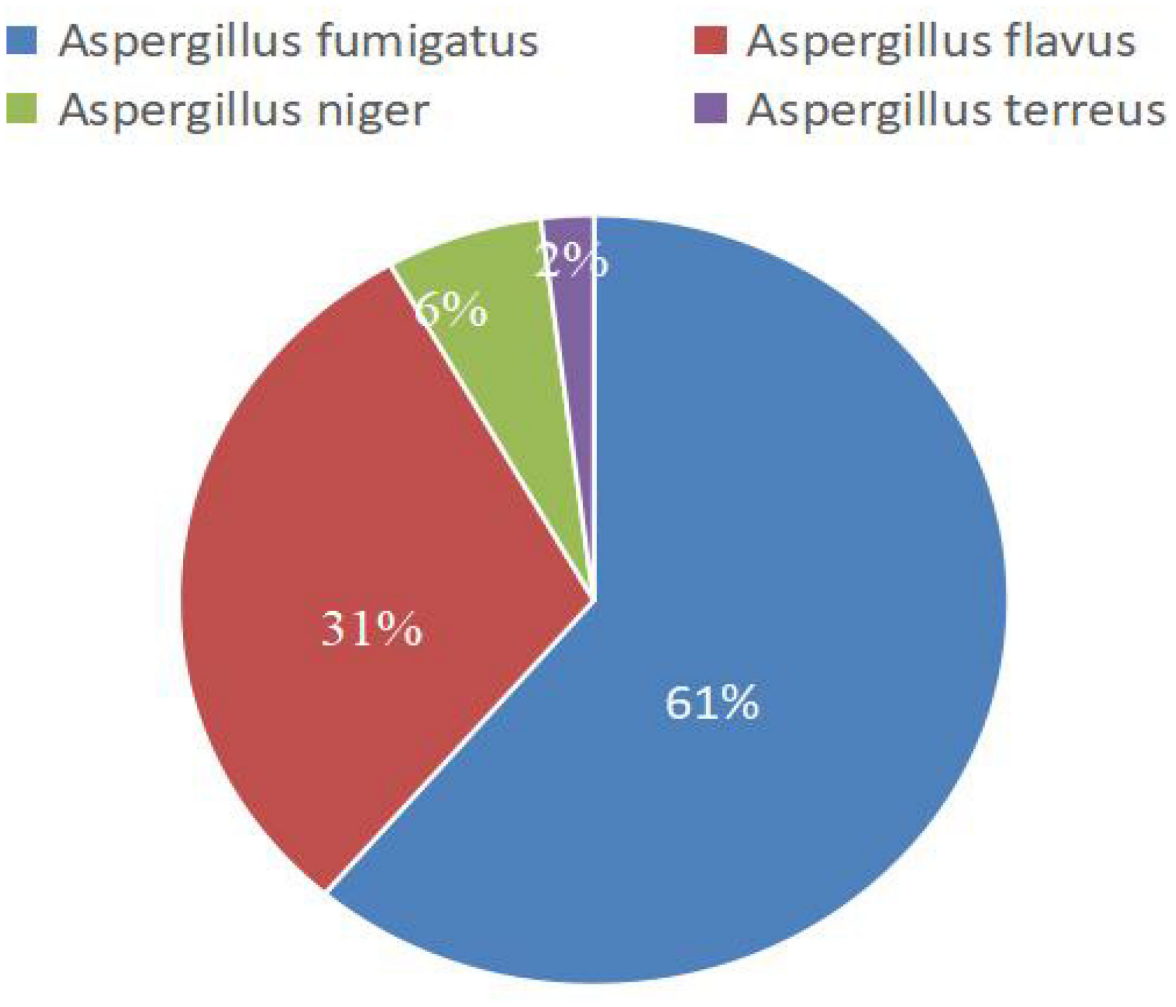
The proportions of various types of *Aspergillus* detected by mNGS.

mNGS detected 49 strains encompassing various *Aspergillus* species, whereas traditional culture identified 22 fungal strains. **Table 2** illustrates the distribution of these various Aspergillus species, and **Figure 2** shows the composition ratios of these different Aspergillus species..

**Table 2:** Classification of *Aspergillus* detected through mNGS and traditional fungal culture.

Aspergillus species	mNGS	traditional culture
*Aspergillus fumigatus*	30	14
*Aspergillus flavus*	15	7
*Aspergillus niger*	3	1
*Aspergillus terreus*	1	0

### Comparison of Sensitivity, Specificity, PPV, NPV, and J-index among fungal culture, serum GM test, BALF GM test, mNGS, and combination test

**Figure 3:**
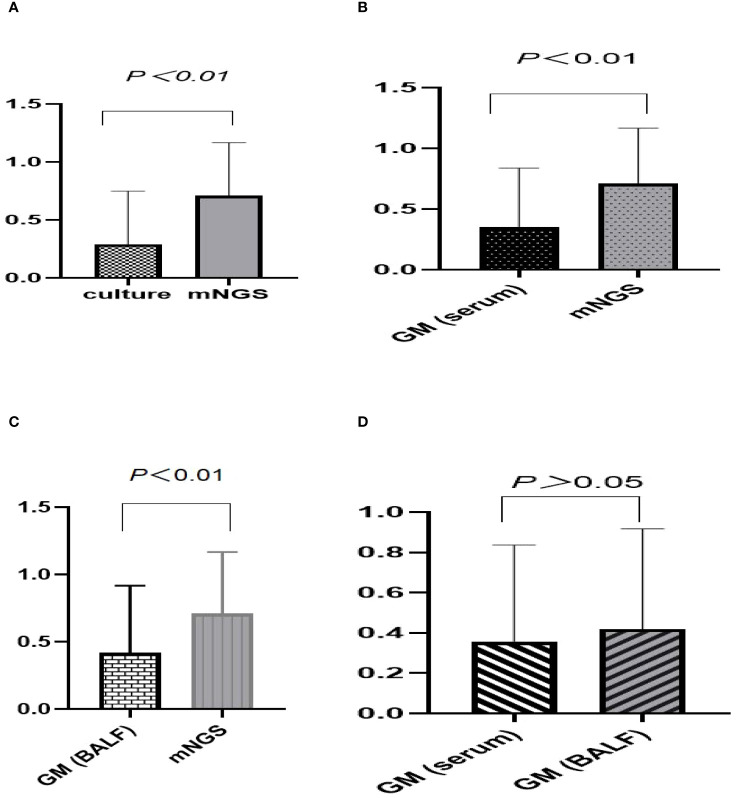
Comparative analysis of sensitivity across various diagnostic methods for suspected IPA. **(A–C)** The sensitivity of mNGS was 70.9%, higher than that of traditional fungal culture (29.0%, *P*<0.01), serum GM test (35.4%, *P*<0.01) and BALF GM test (41.9%, *P*<0.01). **(D)** The sensitivity of BALF GM testing (41.9%) exceeds that of serum GM testing (35.4%), with no statistically significant difference between the two groups (*P*>0.05).

We compared the differences in sensitivity, specificity, positive predictive value, negative predictive value, and J-index among fungal culture, serum GM test, BALF GM test, mNGS, and combination test (**Table 3**). The sensitivity of mNGS for diagnosing IPA was 70.9%, higher than that of traditional fungal culture (29.0%), serum GM test (35.4%) and BALF GM test (41.9%) (*P*<0.05) (**Figures 3A–C**). The sensitivity of BALF GM testing (41.9%) exceeds that of serum GM testing (35.4%), with no statistically significant difference between the two groups(*P>*0.05) (**Figure 3D**). When mNGS was combined with the GM test, and fungal culture, the sensitivity increased to 80.6%, surpassing that of any single method.

**Table 3:** Comparison of sensitivity, specificity, PPV, NPV, and J-index among fungal culture, serum GM test, BALF GM test, mNGS, and combination test.

Method	IPA group (n)	Non-IPA group(n)	Sensitivity%	Specificity%	PPV(%)	NPV(%)	J-index
Culture			29.0(18/62)	90.6(58/64)	75.0	56.8	19.6
pos	18	6					
neg	44	58					
GM (serum)			35.4(22/62)	87.5(56/64)	73.3	58.3	22.9
pos	22	8					
neg	40	56					
GM (BALF)			41.9(26/62)	85.9(55/64)	74.2	60.4	27.8
pos	26	9					
neg	36	55					
mNGS			70.9(44/62)	71.8(46/64)	75.8	73.5	42.7
pos	44	18					
neg	18	46					
Combination test			80.6(50/62)	92.2(59/64)	90.9	83.1	72.8
pos	50	5					
neg	12	59					

### Comparison of the diagnostic efficacy of mNGS, fungal culture, the GM test, and combination testing

**Figure 4:**
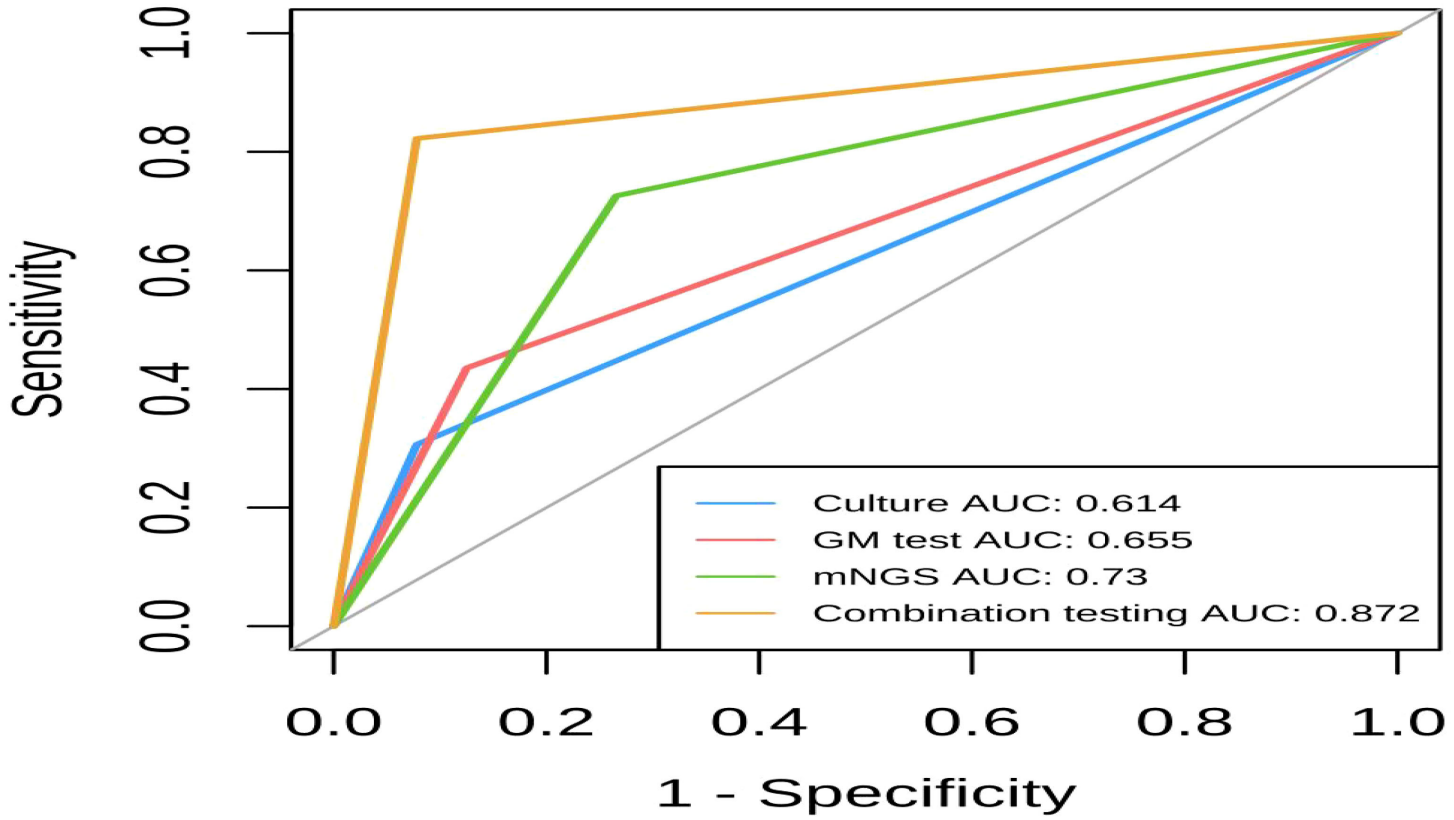
Comparison of different diagnostic methods through the ROC curve.

The area under the ROC curves (AUC) for combined detection, mNGS, GM, and conventional culture were 0.872, 0.73, 0.655, and 0.614, respectively. This suggests that mNGS has a higher diagnostic value for IPA compared to GM and culture alone, and combination testing has the highest diagnostic value for IPA. **Figure 4** illustrates the comparative diagnostic efficacy of mNGS, fungal culture, GM test (BALF), and combination testing.

### Comparison of detection time

The detection time of mNGS proved shorter compared to traditional fungal culture and GM test (*P* < 0.05). **Table 4** compares the detection times of mNGS, fungal culture, and GM testing.

**Table 4:** Comparison of detection time of mNGS, culture, and GM testing.

Examination items	Reporting time(h)
mNGS	19.55 ± 1.23
Culture	78.85 ± 3.56
GM test	27.46 ± 1.98
*P*	<0.05

The difference was significant between culture and mNGS (*P* < 0.05).

The difference was significant between BALF GM test and culture (*P* < 0.05).

## Discussion

*Aspergillus* is widespread in nature and can be found in moldy grains, feed, water, soil, clothing, and furniture. For healthy individuals with normal immune function, ciliary movement in the airways, and phagocytosis by immune cells effectively eliminate inhaled *Aspergillus* fungi (Yang et al., 2022). Despite the daily inhalation of *Aspergillus* spores, *Aspergillus* infections are rare. On the contrary, for individuals receiving immunosuppressive therapy, neutropenic following bone marrow transplantation, or undergoing chemotherapy, these individuals have weakened immune function, making them susceptible to IPA.

Recently, patients with AECOPD in the ICU have been identified as a potential high-risk population for IPA. On the one hand, declining lung function, prolonged exposure to chronic hypoxia, reduced quantity of airway epithelial cilia, and impaired ciliary motility diminish the airway’s defense and clearance mechanisms against *Aspergillus*, facilitating *Aspergillus* colonization (Niu and Zhao, 2022). On the other hand, the use of corticosteroids and antibiotics, mechanical ventilation, central venous catheterization, inadequate nutritional intake, and other measures heighten the risk of developing IPA (Chen et al., 2022).

Currently, in all patients with AECOPD, particularly those admitted to the ICU, suspicion of IPA should be raised when presenting with fever refractory to antibiotic treatment, anomalous chest CT scans, and evidence of fungal elements in respiratory samples, AECOPD complicated by IPA is associated with a mortality rate exceeding 50%, emphasizing the crucial importance of early and accurate diagnosis. However, owing to the lack of specific clinical manifestations and precise early diagnostic tools, discerning IPA from bacterial infections, other fungal infections, and malignancies can prove challenging at times (Guinea et al., 2010).

Presently, traditional methods for detecting *Aspergillus* encompass fungal culture, the G test, the GM test, and antibody detection techniques. Fungal culture serves as the fundamental diagnostic method for IPA, enabling species identification and *in vitro* drug sensitivity testing of fungal pathogens. Conventional microbial culture relies on the growth rate of live microorganisms, with the culture period for common bacterial pathogens typically requiring at least 8 hours and for fungi at least 5 days (Molinos-Castro et al., 2020). In contrast, mNGS provides test results within 24 hours of sample collection, significantly shortening the detection time. According to various studies, the positive rate of fungal culture in respiratory samples from patients with IPA varies from 11.8% to 50% (Hammond et al., 2020). However, *Aspergillus* colonization in the airways may lead to false-negative or false-positive results. In this study, the sensitivity of respiratory sample culture for diagnosing IPA was only 29%, significantly lower than that of mNGS. GM, present on the cell walls of various fungi, is released into the blood and other body fluids during hyphal growth and is commonly utilized for early screening and continuous monitoring of *Aspergillus* infections. GM serves as a reliable serum marker for clinical adjunctive diagnosis of IPA. Continuous monitoring of serum GM levels in high-risk patients, such as COPD, tumors, and organ transplant recipients, facilitates the early identification and recognition of infections (Everaerts et al., 2018; Diao et al., 2022). In immunocompetent patients, neutrophils impede *Aspergillus* entry into the bloodstream and can engulf GM antigens, thereby diminishing the sensitivity of serum GM testing (Huang et al., 2020). A study revealed that in patients lacking neutropenia, the sensitivity of BALF GM detection reaches 87%, whereas serum GM detection yields a sensitivity of only 42% (Jenks et al., 2020).Our experiment suggests that the sensitivity of BALF GM testing (41.9%) for diagnosing IPA surpasses that of serum GM testing (35.4%), with no statistically significant difference between the two groups, possibly due to prior antifungal therapy received by the patients.

Additionally, the definitive diagnosis of IPA necessitates a lung biopsy demonstrating invasive growth and yielding positive culture (Xie et al., 2019). However, conducting such biopsies in ICU patients with AECOPD often poses significant risks due to hemodynamic and respiratory instability, along with potential coagulation disorders (Miao et al., 2018; Chen et al., 2021). Therefore, clinicians frequently encounter challenges in obtaining adequate biopsy samples from the infection site, thereby amplifying the risk of false-negative results (Wu et al., 2020).

In such circumstances, the importance of developing and implementing faster and more accurate detection methods is highlighted, mNGS technology demonstrates unique advantages (Zhu et al., 2023). mNGS, a high-throughput DNA sequencing technique, enables rapid and comprehensive pathogen detection without requiring primers or probes. It identifies the genomes of all microorganisms, including bacteria, viruses, and fungi, irrespective of prior knowledge of microbial species in the sample (Zhan et al., 2021). This renders mNGS advantageous in diagnosing complex infections. It is undeniable that NGS has received considerable attention in the field of respiratory infections and fundamentally transformed our comprehension of the airway (Kim et al., 2021).

However, despite numerous studies reporting the clinical applications of mNGS, most of them have summarized retrospective analyses of various patient populations. Moreover, there are fewer reports on the utilization of mNGS in early diagnosis of IPA. Some studies have shown that in patients with neutropenia, the sensitivity of mNGS for diagnosing IPA in BALF is 92.31%, higher than traditional detection methods or GM testing. The AUC of BALF mNGS is 0.925, indicating good performance. In the diagnosis of invasive fungal infections (IFIs), the ROC curve indicates that the performance of mNGS (particularly lg(RPKM)) surpasses that of CMTs (Deng et al., 2023; Jia et al., 2023).In this study, we collected data from patients with AECOPD admitted to the ICU. Most of these patients exhibited characteristics such as long-term use of antibiotics and steroids, mechanical ventilation, central venous catheterization, and risk of malnutrition, all of which increase the risk of invasive aspergillosis infection. Through analysis, the diagnostic sensitivity of BALF mNGS was found to be 70.9%, higher than that of the BALF GM test (41.9%), serum GM test (35.4%) and traditional fungal culture (29.0%). BALF mNGS exhibited higher sensitivity and a shorter detection time, showing promise for improving treatment effectiveness in patients with AECOPD, however, its specificity falls below that of conventional culture methods.

This study also has some limitations. Firstly, it is a single-center study with a small sample size, potentially limiting its generalizability to a broader population. Consequently, the findings primarily reflect the detection performance of mNGS within a subset of patients from a single hospital (Wang et al., 2022; Shi et al., 2023). Future research should aim for multicenter mNGS testing to address the insufficient sample size. Secondly, the interpretation of mNGS results lacks a universally recognized standard, necessitating physicians to integrate patients’ medical histories, clinical presentations, and other laboratory test results to arrive at a final clinical diagnosis. This process often entails subjective judgment. Moreover, the high cost associated with mNGS limits its widespread implementation and imposes a significant financial burden on patients to some extent.

## Data availability statement

The original contributions presented in the study are included in the article/supplementary material. Further inquiries can be directed to the corresponding author.

## Ethics statement

The studies involving humans were approved by The Ethics Committee of Xinxiang City Central Hospital, Henan Province, China. The studies were conducted in accordance with the local legislation and institutional requirements. The ethics committee/institutional review board waived the requirement of written informed consent for participation from the participants or the participants’ legal guardians/next of kin because Since retrospective studies primarily involve the utilization of existing data or records without actively intervening with patients or collecting new data, the requirement for written informed consent is typically waived. Written informed consent was not obtained from the individual(s) for the publication of any potentially identifiable images or data included in this article because Since retrospective studies primarily involve the utilization of existing data or records without actively intervening with patients or collecting new data, the requirement for written informed consent is typically waived.

## Author contributions

SN: Writing – original draft, Writing – review & editing. DL: Conceptualization, Data curation, Formal analysis, Funding acquisition, Investigation, Methodology, Project administration, Resources, Software, Supervision, Validation, Visualization, Writing – review & editing. YY: Writing – original draft. LZ: Writing – original draft, Conceptualization, Data curation, Writing – review & editing.
